# Re-acidification of limed soil alters aggregate stability and soil carbon turnover in an agroecosystem

**DOI:** 10.1039/d6ra01955b

**Published:** 2026-07-02

**Authors:** Ameer Hamza, Danutė Karčauskienė, Ieva Mockevičienė, Regina Repšienė, Noor-Us-Sabah, Alvyra Šlepetienė

**Affiliations:** a Vezaiciai Branch, Institute of Agriculture, Lithuanian Research Center for Agriculture and Forestry Klaipeda 96219 Lithuania danute.karcauskiene@lammc.lt; b Department of Soil & Environmental Science, College of Agriculture, University of Sargodha 40040 Pakistan; c Chemical Research Laboratory, Institute of Agriculture, Lithuanian Research Center for Agriculture and Forestry Instituto al. 1, Kedainiai District LT-58344 Akademija Lithuania

## Abstract

Soil acidification adversely influences the soil structural stability and carbon sequestration. The current study exploited a unique 75 year liming experiment established in 1949 on Retisols in Lithuania to evaluate how historically generated soil pH gradients (pH 3.82, 3.98, and 5.52), maintained after 19 years without liming (2005–2024), influence soil aggregate stability and aggregate-associated carbon and nutrient pools. Soil samples were collected from 0–10 and 10–20 cm depths and separated into four aggregate size classes: macroaggregates (MACA, 8.00–2.00 mm); mesoaggregates (MESA, 0.630–0.200 mm); microaggregates (MICA, 0.063 mm); and the silt–clay fraction (SCF, <0.063 mm) by dry sieving. Increasing the soil pH from 3.82 to 5.52 substantially improved the soil structural integrity at both soil depths. Under pH 5.52, the proportion of MACA increased from 45.63% to 52.45% at 0–10 cm and from 46.86% to 55.13% at 10–20 cm. The highest mean weight diameter (2.78 and 2.99 mm) and geometric mean weight (1.17 and 1.19 mm) were recorded under pH 5.52 in surface and subsurface soils, respectively. Soil organic carbon increased significantly across all aggregate fractions with increasing pH, with the maximum SOC concentration (22.95 g kg^−1^) observed in the SCF at pH 5.52 in the surface soil. Permanganate-oxidizable carbon showed the strongest response in MACA, increasing by 54.66% at pH 5.52 compared with strongly acidic soil (pH 3.82). In contrast, water-extractable organic carbon was consistently highest in MICA, reaching 333.66 mg kg^−1^ at pH 5.52. Total nitrogen and total phosphorus also increased significantly with increasing pH across aggregate fractions and soil depths. Correlation analysis revealed strong positive associations between soil pH, aggregate stability, and carbon and nutrient pools. Conclusively, increasing soil pH to moderately acidic conditions (5.52) promoted MACA formation, enhanced aggregate stability, and increased both stable and labile carbon pools, highlighting long-term liming as an effective strategy for restoring soil structure and improving carbon and nutrient retention in Retisols.

## Abstract 2

Liming legacy improved aggregate stability in re-acidified Retisols. Higher soil pH enhanced stable and labile soil carbon pools. Soil pH increased nitrogen and phosphorus retention across aggregates.

## Graphical abstract

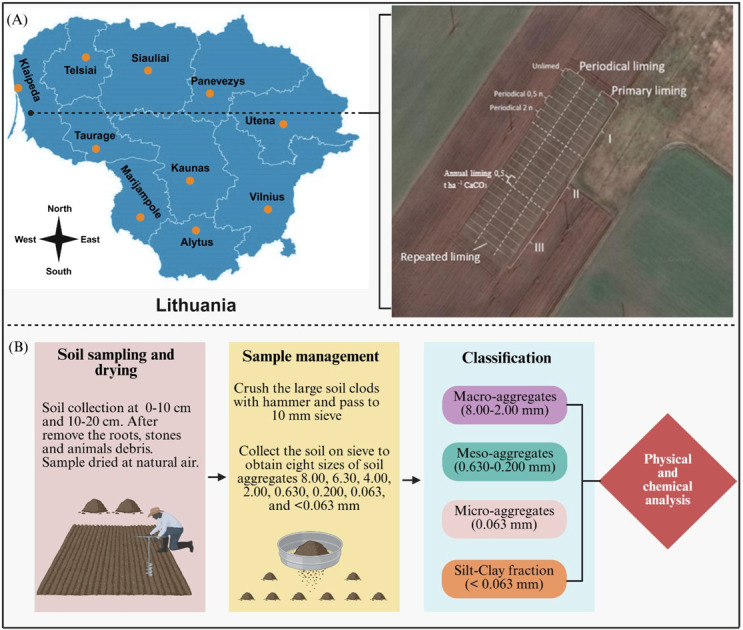

## 1 Introduction

Soil acidification is a biogeochemical process that takes place concurrently with soil formation and evolution. It may require decades or centuries to become discernible in natural conditions.^1^ It has been reported that acid soils cover about 50 percent of the world's arable land.^2^ Progressive acidification and re-acidification lead to the accumulation of aluminium (Al^3+^) and loss of essential nutrients, inhibition of microbial activity, and eventual degradation of soil structure and organic matter (OM) dynamics. This has become a growing menace to agricultural production worldwide.^3,4^ The post-liming drop in pH can change the cation exchange equilibrium, decrease the interactions between Ca^2+^ and organic matter, while reducing the cation bridging processes that promote aggregate stabilization.^5–7^ Polyvalent cations, which include Ca^2+^ and Mg^2+^ ions, are key agents of flocculation of clay particles and facilitation of interactions between surfaces on minerals and organic matter.^8,9^ When the soil pH gets low, these stabilizing mechanisms can also decline, and this can have an impact on aggregate stability and soil organic carbon (SOC) protection in aggregates and nutrient pools, as they directly impact soil fertility, resilience, and long-term productivity.^10^

Aggregate stability is a major indicator of the physical health of soil. Soil aggregates are secondary structures formed from mineral particles through the combined influence of organic and inorganic substances.^11,12^ Their formation depends on various factors, such as environmental conditions, soil management techniques, and the properties of the plants and soil.^13,14^ Stable aggregates preserve the organic carbon in the soil and prevent the rapid mineralization of soil organic carbon, stabilize the pore connectivity, and improve nutrient retention.^15^ On the other hand, aggregate breakdown increases the rate of carbon loss and interferes with the nutrient cycling process, especially in acidic soils^16^ where the biological activity is already limited. As a result, the viable measures to counter soil acidification play key roles in the maintenance of soil functioning in agroecosystems. The soil management practices may also change the soil acidity, exchangeable cation content and composition, content of humus-forming agents, and biological activity, and change the rate and direction of the humification processes.^17–19^

Although it is important, little attention has been paid to the reaction of aggregate stability and concomitant carbon and nutrient pools to long-term pH changes after the cessation of liming. The Ca^2+^ addition in the presence of lime helps stabilize SOC.^20^ The reaction between SOC and mineral particles through polyvalent cations is one of the most essential ways of enhancing SOC protection and storage in acidic soils.^21^ In a bid to control the SOC of the acidic soils, methods of curbing the acidic soils, countering the toxic elements in the soil, and encouraging polyvalent cations like Ca^2+^ and Mg^2+^ across the soil profile are vital. Various long-term studies have demonstrated the potential of surface lime application to enhance the storage and accumulation of SOC in acid soil through increasing the growth of plants and SOC input.^22,23^ Nonetheless, some scientific findings suggest that lime usage improves SOC mineralization, particularly on acid soil.

Liming, which portrays a promising capability of raising the pH of soil and enhancing the production of crops, has been utilized as a common practice of acidification amelioration.^24–26^ Liming displayed deep influences on biogeochemical processes in the carbon (C) cycling, such as SOC mineralization.^27,28^ Liming increases soil pH, calcium, and flocculates clay particles, increases the formation and stability of aggregates, and stimulates the activity of microorganisms involved in the transformation of organic matter.^29^ Previous studies have shown that aggregate stability and soil organic carbon improve with liming, but the long-term persistence following periods of liming, especially following the cessation of liming, is not well comprehended.

The current study exploits a unique long-term liming experiment established in 1949, which involved primary, repeated, and periodic liming until 2005, followed by nearly two decades (2005–2024) without further lime application. This management history generated persistent and contrasting soil pH levels (3.82, 3.98, and 5.52), providing a rare opportunity to examine the long-term legacy effects of liming under conditions of progressive re-acidification. The resulting soil pH gradients represent long-term pedogenic and management-induced trajectories, providing an opportunity to assess how historical liming legacy influences present-day aggregate stability, carbon turnover, and nutrient retention. The specific objectives for this study were to: (i) assess how historically induced soil pH gradients influence soil aggregate size distribution and aggregate stability nearly 20 years after liming cessation; (ii) quantify the responses of aggregate-associated carbon fractions like SOC, permanganate oxidizable carbon (POXC), water extractable organic carbon (WEOC), nutrient pools total nitrogen (TN) and total phosphorus (TP) across soil depths under contrasting pH conditions; and (iii) to elucidate the relationship between soil pH, aggregate stability, and carbon-nutrient stabilization in Retisols. These findings will be helpful to develop strategies to achieve well-developed soil health, carbon sequestration, and nutrient retention in acidic soils.

## 2 Materials and methods

### 2.1 Experimental site and climatic conditions

**Fig. 1:**
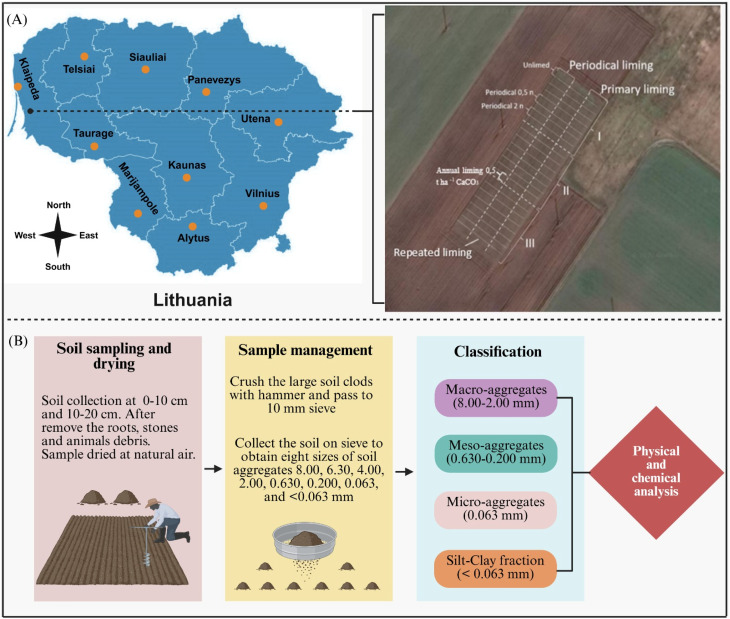
Geographic location of the long-term experimental site in Lithuania (A) and sampling process of soil for physical and chemical analysis (B).

In 1949, a long-term field experiment was initiated on moraine loam soil (Bathygleyic Dystric Glossic Retisols) at the Vezaiciai branch (55°43′ N, 21°27′ E) of the Institute of Agriculture, Lithuanian Research Centre of Agriculture and Forestry (LAMMC). The location of the experimental site is shown in Fig. 1A. The soil contains 51.10%, 33.6%, and 15.3% as sand, silt, and clay fractions, respectively, while the basic properties of bulk soil, such as organic carbon (OC), permanganate oxidizable carbon (POXC), water extractable carbon (WEOC), total nitrogen (TN), and total phosphorus (TP) are shown in Table 1. The research site is in a cold, humid climate zone with an average annual rainfall of 938 mm, and a mean annual air temperature of 7.86 °C, calculated for the 2005–2024 climate normal period based on data from meteorological data from Vėžaičiai automatic station. The climate of this area is strongly maritime, and soil acidification is a natural process that is enhanced by anthropogenic activities. The complete cropping history of the experimental site is depicted in Table 2.

**Table 1:** Bulk soil properties measured at the sampling time under re-acidification following long-term liming at two depths (0–10 and 10–20 cm)

Depth	Treatment	SOC (mg kg^−1^)	POXC (mg kg^−1^)	WEOC (mg kg^−1^)	TN (%)	TP (%)
	pH-3.82(CK)	16.47	368.12	242.82	0.125	0.065
0–10 cm	pH-3.98	17.01	398.87	257.28	0.126	0.068
	pH-5.52	20.18	561.19	297.84	0.129	0.072
	pH-3.82(CK)	15.44	305.96	245.14	0.120	0.063
10–20 cm	pH-3.98	16.73	391.77	282.21	0.122	0.065
	pH-5.52	19.03	577.52	298.21	0.129	0.071

**Table 2:** Cropping history of long-term experiment from 1949–2024

Crop rotation pattern from 1949 to 2005	
Crop mixture	Applied fertilizers (N–P–K)
Fodder beet	68–80–120
Barley sown with perennial grasses	60–60–60
Perennial grasses	0–60–60
Perennial grasses	0–60–60
Winter wheat	60–60–60
Pea–barley mixture for grain	0–60–60
Vetch–oat mixture for forage	0–60–60

**In 2008, the long-term experiment was restructured, and the crop rotation pattern changed**	
Barley sown with perennial grasses	60–60–90
Perennial grasses	0–90–90
Winter triticale	90–60–100
Spring rape	120–90–150

**In 2020, the crop rotation was revised as follows**	
Lupins + oats	60–60–60
Triticale + reddish (incorporated)	90–90–100
Oats + perennial grasses (red clover and timothy)	60–60–90
Perennial grasses (red clover and timothy)	0–60–90
Winter wheat	60–60–60

### 2.2 Experimental treatments and design

A long-term experiment was designed to evaluate the effects of lime dust on the physicochemical characteristics of moraine loam soil and carbon accumulation. The experimental design included three treatments: (i) pH = 3.82; (ii) pH = 3.98; (iii) pH = 5.52. Every treatment was repeated thrice with a randomized complete block design (RCBD) and a factorial set-up with plot dimensions of 4.25 × 6.0 m. The experiment was originally established in 1949 as an RCBD with three replicates. The plot positions have remained fixed since establishment. The primary liming was done in 1949 with the following different rates of slaked lime: 0.5 (3.3 t ha^−1^) and 2.0 (13.2 t ha^−1^), according to the hydrolytic acidity of the soil. The primary liming effect can be noticed up to 1964. In 1964, the experiment was divided into two strips. The impact of primary liming was still observed in the first strip. In 1965, the second strip was limed continuously with a rate of 0.5 liming rate according to the hydrolytic acidity of the soil (3.3 t ha^−1^). Periodic liming has commenced on the background of primary and repeated liming in 1985, and since then it has been done using pulverized limestone (92.5% CaCO_3_). Periodic liming at the 0.5 rate was done every 7 years and liming at the 2.0 rate was done every 3–4 years. The lime was spread over the soil on the surface as well as applied to the soil using a cultivator at a depth of 7–12 cm. Until 2005, the soil was periodically limed. The soil was not limed from 2005 to 2024, and the changes in soil parameters were observed. The soil samples analysed in the present study were collected in 2024 from the original experimental plots established in 1949. Therefore, the measured soil properties reflect the cumulative legacy effects of 75 years of contrasting liming histories followed by 19 years of natural re-acidification after liming cessation. The degree of pH decline from the last liming event (2005) to sampling (2024) across treatment is described in Table 3. Using the long-term liming system – primary (1949), repeated (1965), and periodical liming (1985–2005) from 1949 to 2005, various soil pH levels were generated (Table 4).

**Table 3:** Degree of pH decline from the last liming event (2005) to sampling (2024)

Treatments	Years				
	2005	2010	2015	2020	2024
No liming (control)	4.2	4.2	4.1	3.97	3.82
0.5 t ha^−1^ every 7 years	5.60	5.60	5.36	4.60	3.98
2.0 t ha^−1^ every 3–4 years	6.70	6.60	6.43	6.06	5.52

**Table 4:** Scheme and total amounts of CaCO_3_ applying the different intensities of liming

Liming intensity	Total amount of CaCO_3_ applied (t ha^−1^)			Total amount of CaCO_3_ applied t ha^−1^ (1949–2005)	pH_KCl_
	Primary liming, 1949	Repeated liming, 1965	Periodical liming, 1985–2005		
Un-limed	0	0	0	—	4.2
Liming at 0.5 t ha^−1^ of the liming rate every 7 years	3.3	3.4	11.4	18.1	5.6
Liming at 2.0 t ha^−1^ of the liming rates every 3–4 years	13.2	1.7	90.0	104.9	6.7

### 2.3 Collection of soil samples

Soil samples were collected from each plot of the experimental field (Fig. 1B) in spring before wheat sowing in 2024. From each experimental plot, three random sampling points were chosen. An iron sampler with an inner diameter of 10 cm and a length of 10 cm was used to collect six tubes from two depth intervals: the upper soil layer (0–10 cm) and the sublayer (10–20 cm). The samples collected were thoroughly mixed to ensure uniformity. To maintain statistical validity, 18 soil samples were collected (3 treatments × 3 replications × 2 depth layers). All samples were transported to the laboratory and air-dried. The soil clods were gently broken along their natural cleavage planes and used for aggregate classification, carbon, and nutrient analyses.

### 2.4 Experimental protocol

#### 2.4.1 Soil aggregate analysis

For dry sieving of aggregate, air-dried soil samples of 1000 g were weighed and sieved using the Haver EML 450 digital plus (Haver & Boecker 59302 OELDE, Germany) sieve shaker (5 minutes with 1.5 amplitude) with a set of 8.00, 6.30, 4.00, 2.00, 0.630, 0.200, 0.063, and <0.063 mm mesh sizes. The aggregates from dry sieving were grouped into four categories: macroaggregates (MACA, 8.00–2.00 mm); mesoaggregates (MESA, 0.630–0.200 mm); microaggregates (MICA, 0.063 mm); and the silt–clay fraction (SCF, <0.063 mm). The stability of aggregates, including mean weight diameter (MWD) and geometric mean weight (GMW), was expressed in mm and driven by the formula shown in eqs. (1) and (2) (ref. 30 and 31) respectively and estimated by the following eqn (1) and (2).1
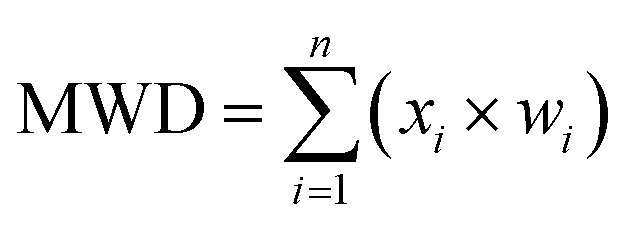
2
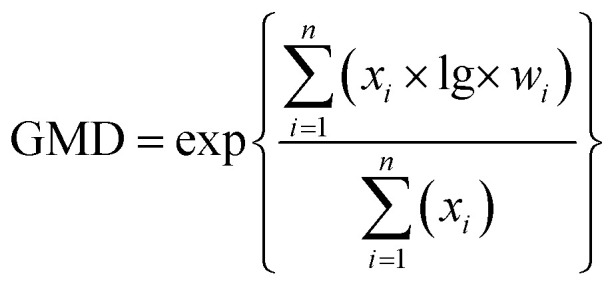
where *n* is the number of aggregate fractions, *x*_*i*_ is the mean diameter of each aggregate fraction, and *w*_*i*_ is the weight of each aggregate fraction.

#### 2.4.2 Soil C, N, and P analysis from aggregate fraction

The chemical analyses were conducted in the Chemical Research Laboratory at the Institute of Agriculture, LAMMC. The following chemical properties of the soil were identified pH measured in 1 M KCl, and mobile aluminum measured by the Sokolov method. To determine soil organic carbon (SOC), permanganate oxidized carbon (POXC), water extractable organic carbon (WEOC), total nitrogen (TN), and total phosphorus (TP), soil aggregate samples were ground and filtered using a 0.25 mm sieve. A spectrophotometric method at 590 nm was used to measure SOC after dichromatic oxidation, and the results were compared with those of glucose as a standard.^32^ The WEOC was established based on the SKALAR methodology. To start with, distilled water was sprayed onto the soil at 1 : 5 ratio, and the suspension was prepared. Then centrifuge the sample at 4500 rpm for 15 minutes and filter. Subsequently, the automatic measurement process was initiated according to the IR detection procedure, followed by UV-catalysed persulfate oxidation under nitrogen (SKALAR, The Netherlands). The method was used to analyse the POXC described by Weil.^33^ In this procedure, 2.5 g of soil was added to 18 mL of deionized water, followed by the addition of 2 mL of KMnO_4_ solution and shaking for 2 minutes. Thereafter, centrifuge at 4800 rpm for 4 minutes, then allow the suspension to rest for 10 minutes. As the samples settle, pre-add 40 mL of DI water to the dilution tube. After 10 minutes, rapidly withdraw 0.5 mL of suspension, add it to the dilution tube, and bring the volume to 50 mL. The POXC value was calculated by measuring the spectrophotometer at 540 nm (ref. 33). The total nitrogen (TN) in the soil was determined using the Kjeldahl method, and the total phosphorus (TP) using the AL method.

### 2.5 Statistical analysis

All observed data were statistically analysed using Statistix 8.1 (Statistix, Tallahassee, FL, USA). A two-way analysis of variance (ANOVA) was conducted to determine significant differences between treatments using LSD's test with a significance level of *p* < 0.05, and the mean of three replicates plus standard error was provided (±S.E.). Pearson's correlation analysis was performed to evaluate the relationships among soil structural and biochemical parameters, with correlation coefficients (*r*) computed based on paired observations and significance assessed at *p* < 0.05 by using BioRender software. Principal component analysis (PCA) was performed using the R statistical software package. The graphical demonstration of the data was done using the Sigmaplot 12.5 (Sigmaplot v13.0.0.83, Systat Software Inc., Chicago, USA) and BioRender Softwares.

## 3 Results

### 3.1 Effects of soil pH on aggregate size distribution

**Fig. 2:**
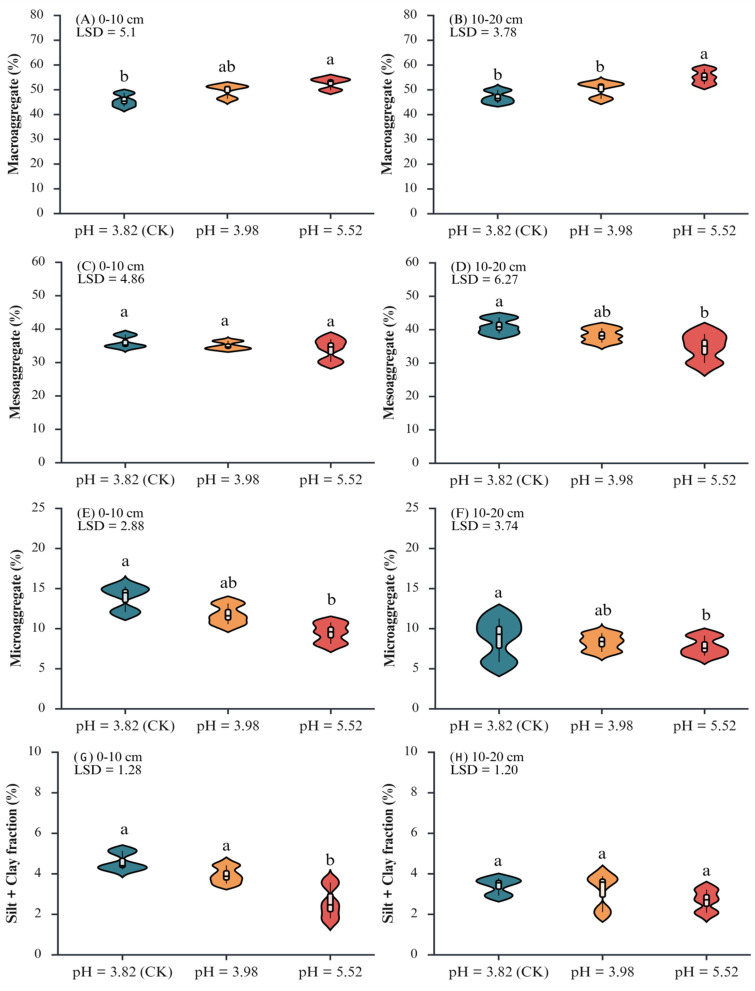
Re-acidification impacts on the distribution of different soil aggregate fractions: (A and B) macroaggregate, (C and D) mesoaggregate, (E and F) microaggregate and (G and H) silt–clay fractions in Retisols at two soil depths (0–10 and 10–20 cm).

Soil pH levels resulting from long-term liming and subsequent liming cessation (2005–2024) significantly influenced the soil aggregate distribution at both soil depths (0–10 cm and 10–20 cm) (Fig. 2A–F). Among the pH levels, MACA proportion increased consistently with increasing soil pH, with the highest MACA (52.45% and 55.13%) proportion observed at pH 5.52 and the lowest (45.63% and 46.86) at pH 3.82 under both 0–10 and 10–20 cm depths (Fig. 2A and B). This pattern was more pronounced in the surface soil (0–10 cm), where differences among pH treatments were clearly expressed. In contrast, soils with lower pH levels (3.82 and 3.98) exhibited a reduced proportion of MACA, indicating weaker aggregate formation under high acidic conditions. In case of MESA distribution, at both depths, effect of soil pH levels was non-significant on MESA distribution (Fig. 2C and D). MICA proportions exhibited an opposite trend to MACA (Fig. 2E and F). The highest MICA 13.89% and 8.74% proportion was recorded at the lowest soil pH 3.82 (control) in 0–10 cm and 10–20 cm soil depths, respectively. Whereas, increasing soil pH resulted in a marked decline in MICA proportion at both depths. The proportion of the SCF fraction was strongly affected by soil pH levels (Fig. 2G and H). Soils having pH 3.82 exhibited a highest SCF fraction (4.56% in 0–10 cm and 3.40% in 10–20 cm depth), while increasing pH resulted in a consistent reduction in SCF.

### 3.2 Effects of soil pH on soil aggregate stability indices

**Fig. 3:**
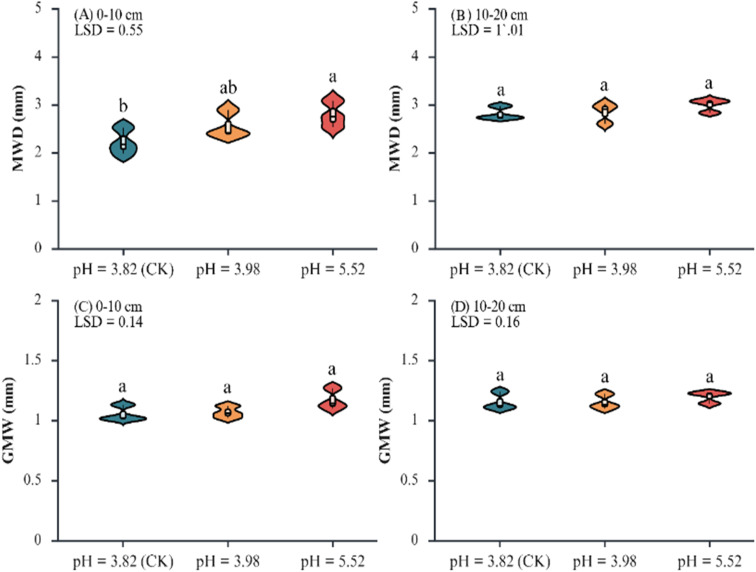
Re-acidification impacts on soil structural stability indices: (A and B) mean weight diameter (MWD) and (C and D) geometric mean weight (GMW) in Retisols at two soil depths (0–10 and 10–20 cm).

Aggregate stability indices, including mean weight diameter (MWD) and geometric mean weight (GMW), increased significantly with increasing soil pH at both depths (Fig. 3A–D). The highest MWD (2.78 mm and 2.99 mm) and GMW (1.17 mm and 1.19 mm) values were observed in 0–10 and 10–20 cm soil depth, respectively under soil pH 5.52. Whereas the lowest values were recorded under strongly acidic conditions (pH 3.82). The response of both indices followed a similar pattern across depths, although absolute values were generally higher in the 0–10 cm layer than in the 10–20 cm soil profile.

### 3.3 Carbon fractions in soil aggregates under different pH levels

**Fig. 4:**
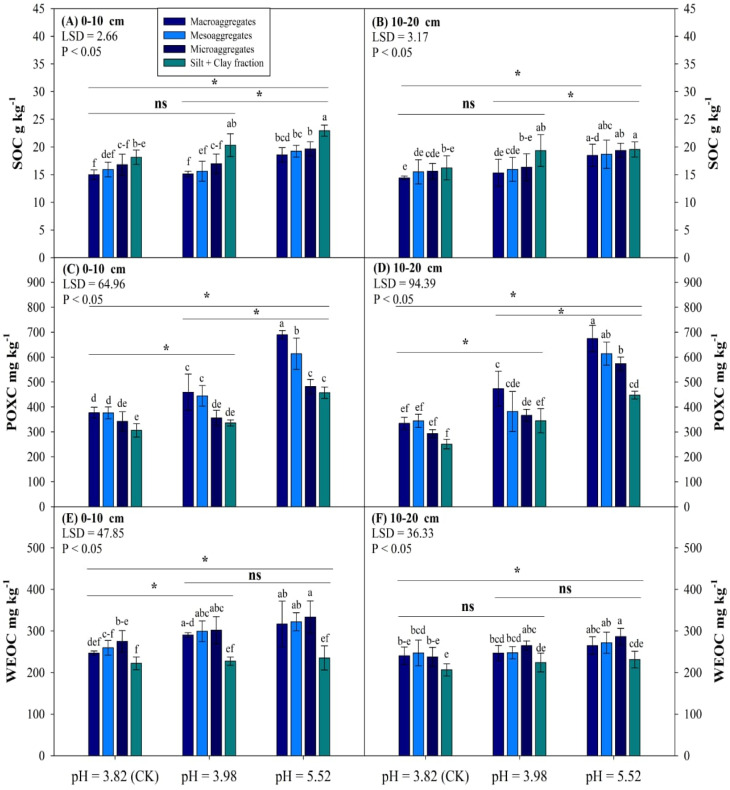
Response of soil carbon fractions to re-acidification: (A and B) SOC (g kg^−1^), (C and D) POXC (mg kg^−1^) and (E and F) WEOC (mg kg^−1^) contents in different aggregate fractions in Retisols at two soil depths (0–10 and 10–20 cm). “*” indicates significant and “ns” shows non-significant differences among pH levels at (*p* < 0.05).

A significant impact of pH levels was observed on soil organic carbon (SOC) content across aggregate fractions and soil depths (Fig. 4A and B). At the 0–10 cm depth, SOC consistently increased with rising soil pH levels among all aggregate fractions. The higher SOC contents were recorded at pH 5.52 in all aggregate fractions, with the highest value (22.95 g kg^−1^) observed in SCF (Fig. 4A). The least SOC contents in all aggregate fractions were observed at pH 3.82 (CK). At 10–20 cm depth, SOC contents were lower than observed at 0–10 cm depth. Under pH 3.82 (CK), SOC ranged from 14.42 g kg^−1^ (MACA) to 16.22 g kg^−1^ (SCF) (Fig. 4B). Increasing soil pH to 5.52 resulted in pronounced SOC enhancement, with highest SOC contents (19.54 g kg^−1^) recorded in SCF, followed by MICA (19.37 g kg^−1^).

Permanganate oxidized carbon (POXC) enhanced significantly with increase in soil pH levels across aggregate fractions and depths (Fig. 4C and D). The content of POXC was 377.0 mg kg^−1^ in the MACA at pH 3.82, while in soil having pH 5.52 had 54.66% higher POXC contents was recorded at 0–10 cm depth. Similarly, at 10–20 cm depth, the highest POXC contents were observed at pH 5.52, with values of 675.00, 614.00, 573.67, and 447.67 mg kg^−1^ in MACA, MESA, MICA, and SCF, respectively.

Water-extractable organic carbon (WEOC) varied significantly among aggregate fractions and soil pH levels (Fig. 4E and F). In the profile from 0 to 10 cm depth WEOC markedly improved at pH 5.52 by giving 333.66 mg kg^−1^ in MICA, followed by MESA (322.22 mg kg^−1^) and MACA (316.79 mg kg^−1^). However, in 10–20 cm soil profile, the highest WEOC contents were observed under pH 5.52, particularly in MICA (286.50 mg kg^−1^) and least was observed at pH 3.82. Under both soil profiles, MICA consistently showed higher WEOC contents than MACA, MESA and SCF.

### 3.4 Nitrogen and phosphorus in soil aggregates under different pH levels

**Fig. 5:**
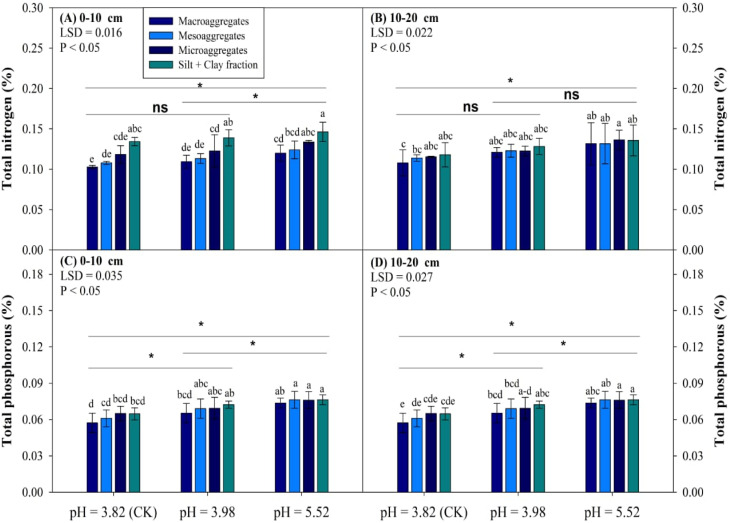
Response of nutrient pool to re-acidification: (A and B) total nitrogen (%) and (C and D) total phosphorus (%) contents in different aggregate fractions in Retisols at two soil depths (0–10 and 10–20 cm). “*” indicates significant and “ns” shows non-significant differences among pH levels at (*p* < 0.05).

The data of the current study showed that pH levels significantly changed the total nitrogen (TN) and total phosphorus (TP) of both soil profiles (Fig. 5A–D). At 0–10 cm depth, as compared to control (pH 3.82), the pH 5.52 produced significantly higher TN in all aggregate fraction. However, among aggregate fractions, the highest TN (0.1463%) was recorded in SCF under pH 5.52, followed by pH 3.98 and pH 3.82. Likely, in the 10–20 cm depth, TN contents were slightly lower than 0–10 cm depth (Fig. 5A and B). The highest TN values were recorded at pH 5.52, particularly in MICA (0.1363%) and MESA (0.1317%) and the least was observed at pH 3.82.

In case of TP content, a consistent increase was observed with increasing soil pH in all aggregate fractions at both soil depths (Fig. 5C and D). At 0–10 cm, TP values increased from 0.0573–0.0650% under pH 3.82 to 0.0737–0.0763% under pH 5.52 in reported aggregate fractions. The highest TP concentrations were observed in MESA and SCF at pH 5.52. Similar trend was noted at 10–20 cm depth, with values ranging from 0.0567–0.0650% in the control (pH 3.82) to 0.0730–0.0757% at pH 5.52. Overall, TP distribution was relatively uniform among aggregate fractions but consistently higher under high pH levels.

### 3.5 Principal component analysis (PCA)

**Fig. 6:**
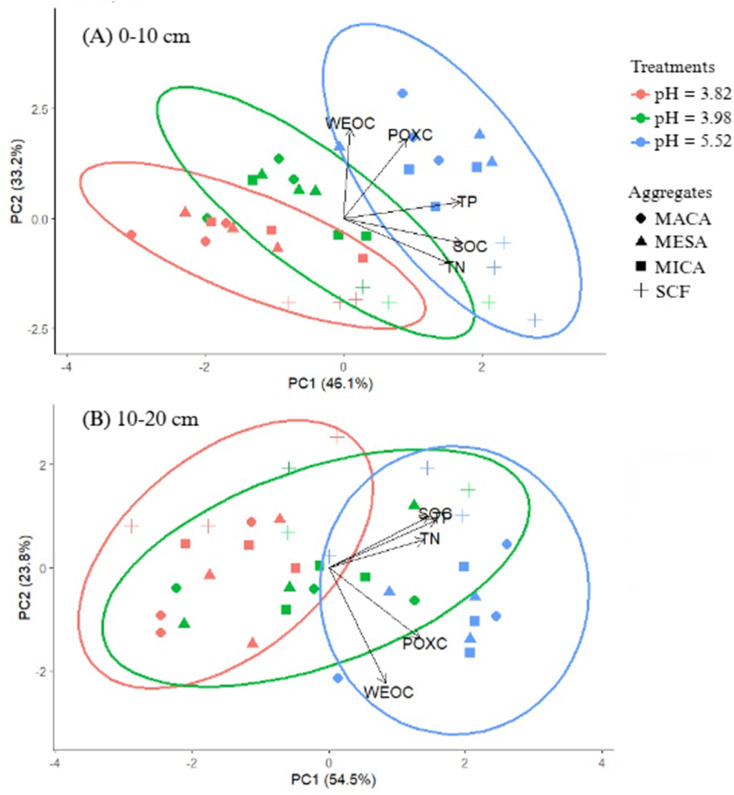
PCA-biplot of soil aggregate-associated carbon and nutrient pools under re-acidification, (A) 0–10 cm and (B) 10–20 cm soil depths. MACA = macroaggregates, MESA = mesoaggregates, MICA = microaggregates SCF = silt–clay fraction, SOC = soil organic carbon, POXC = permanganate-oxidizable carbon, WEOC = water-extractable organic carbon, TP = total nitrogen, and TP = total phosphorus.

It revealed clear depth-dependent associations between soil aggregate fractions and carbon and nutrient pools under varying soil pH conditions (Fig. 6). At 0–10 cm soil layer (Fig. 6A), MACA and MESA clustered closely with labile carbon fractions, particularly POXC and WEOC. This pattern indicates preferential association of biologically active carbon with larger aggregate fractions in surface soils. In contrast, MICA and SCF aligned more strongly with SOC and TP. At 10–20 cm depth (Fig. 6B), PCA ordination showed a marked shift in variable associations. Labile carbon fractions contributed less to the separation of aggregate classes, while SOC and TN were more strongly associated with MICA and SCF. However, MACA displayed weaker relationships with POXC and WEOC at this depth, reflecting reduced coupling between aggregate turnover and labile carbon pools in subsoil layers. Overall, PCA results demonstrate that soil pH modulates aggregate-associated carbon and nutrient pools in a depth-specific manner, with surface soils dominated by labile carbon retention in larger aggregates, and subsurface soils characterized by stronger organo-mineral associations in finer fractions.

### 3.6 Correlation analysis

**Fig. 7:**
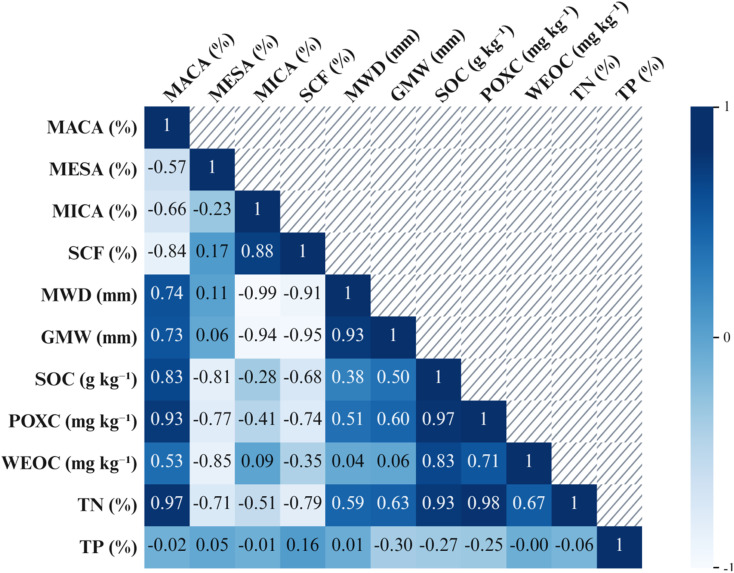
Pearson correlation heatmap between soil aggregate distributions, stability and aggregate-associated carbon and nutrient pools under re-acidification. Dark bule circles denote positive correlations and light blue circles indicate negative correlations. MACA = macroaggregates, MESA = mesoaggregates, MICA = microaggregates SCF = silt–clay fraction, MWD = mean weight diameter, GMW = geometric mean weight, SOC = soil organic carbon, POXC = permanganate-oxidizable carbon, WEOC = water-extractable organic carbon, TP = total nitrogen and TP = total phosphorus.

Pearson correlation analysis (Fig. 7) revealed a strong association among soil structural and biochemical parameters in all reported pH levels (Fig. 6). SOC, POXC, WEOC, TN, and TP generally exhibited positive correlations with soil pH, indicating coordinated responses of carbon and nutrient pools to long-term liming. SOC showed strong positive correlations with TN and TP across aggregate fractions, while POXC and WEOC were closely associated with aggregate size classes, particularly MACA and MICA. Negative correlations were primarily observed between MICA fraction and carbon pools, highlighting contrasting carbon dynamics among aggregate sizes.

## 4 Discussion

Soil acidification has been a global environmental concern^34^ and posing a notable threat to soil productivity.^22^ In the present study, the observed soil pH gradient (3.82, 3.98 and 5.52) can be directly interpreted as representing distinct stages along a re-acidification trajectory following historical liming, where pH 5.52 reflects the least re-acidified condition, pH 3.98 an intermediate re-acidification stage, and pH 3.82 the most advanced re-acidified stage. Our results show that by increasing the soil pH from 3.82 to 5.52, a consistent increase in macroaggregate (MACA) proportion, accompanied by a decline in microaggregates (MICA) and silt + clay fractions (SCF). This structural transition suggests that progressive re-acidification shifts the soil from a stable macroaggregate-dominated system toward a fragmented microaggregate- and SCF-dominated condition. Although Al^3+^ may promote clay flocculation under acidic conditions, but elevated concentrations of exchangeable Al^3+^ and H^+^ promote clay dispersion and inhibit the formation of stable organo-mineral complexes.^35^ Aluminium toxicity disrupts microbial activity and root growth, reducing the input of binding agents such as polysaccharides and fine roots that are essential for MACA formation.^36^ Consequently, soil structure under highly acidic conditions is dominated by MICA and SCF, which represent a less stable structural state. Therefore, the lowest pH treatment (3.82) likely represents the later stage of re-acidification where liming-induced structural benefits have substantially deteriorated. As soil pH increased to moderately acidic levels (up to 5.52), reduced exchangeable Al^3+^ and proton activity enhanced clay flocculation and cation bridging,^4^ mainly *via* Ca^2+^ and Mg^2+^ from liming, that facilitate the hierarchical assembly of MICA into MACA. Simultaneously, higher pH stimulated microbial activity and root growth, promoting extracellular binding agents, particle cohesion, and incorporation of SCF into stable MACA. In contrast, the intermediate pH level (3.98) appears to reflect a transitional stage of re-acidification in which some liming legacy effects persist, but aggregate destabilization has already begun. Previous research discovery revealed that the percentage of MACA in dark brown soil increased significantly when regulated by organic fertilizers, which tended to elevate soil pH, indicating a positive correlation between greater pH and MACA formation.^37^ On the other hand, in one study, it was seen that the higher the soil pH, the lower the fraction of MACA, and higher pH levels may diminish the dispersity of soil particles, consequently influencing the dynamics of MACA.^38^ Nevertheless, the changes in pH on MACA are not always beneficial. For example, in a long-term fertilization experiment, the soil PH reduction was found to influence the MACA stability indirectly through the changes to soil structure.^39,40^ In the present study, the greater aggregate stability indices (MWD and GMW) in pH 5.52 at both soil depths is mainly attributed to the increased disintegration and dissipation of aggregates in the regime of fast wetting, which are aggravated by low pH, resulting in a higher proportion of small-particle-size aggregates and subsequent reduction in the large-particle-size aggregates.^41,42^ Soil aggregate stability is also strongly associated with the existence of polyvalent cations such as Ca^2+^, which are more successful in facilitating soil aggregates. Such a connection implies that the state of soil pH that determines cation exchange capacity and the availability of these cations is a determinant in aggregate stability.^43^

Our findings showed that SOC improved substantially in all aggregate fractions with rising soil pH that emphasizes the importance of soil acidity in controlling carbon storage and stabilization. From a re-acidification perspective, the gradual decline in SOC from pH 5.52 to 3.82 indicates the progressive loss of liming-induced carbon stabilization mechanisms over time. The increase in the SOC concentration in MACA at pH values below 5.52 indicates an increase in the physical protection of organic matter in stable aggregates, which makes them less accessible to microbes and slows down the mineralization.^44,45^ This proves the idea that aggregate hierarchy has a strong control on SOC persistence, especially in structurally enhanced soils. Notably, the most significant SOC contents were always registered in the SCF, particularly at elevated pH levels (3.98 and 5.52). It means that liming not only stimulates macro-aggregation but also contributes to stabilization of carbon by mineral-associated organic matter,^46,47^ which is a well-known long-term carbon pool. Accordingly, soils at the early re-acidification stage (pH 5.52) retained stronger aggregate-associated and mineral-associated carbon protection, whereas advanced re-acidification (pH 3.82) weakened both mechanisms. The simultaneous increase of aggregate-protected and mineral-associated SOC at increased pH supports the complexity of the role played by liming legacy in long-term carbon sequestration.^48^ The^49^ also reported similar findings by stating that liming the soil greatly increased SOC, especially when the pH was low at less than 4.39. Correspondingly, a meta-analysis study found that organic amendments that tend to change soil pH augmented the SOC of all soil aggregate sizes by 16–23%, and the most substantial effects were in soils with pH lower than 6.0.^50^

In the present study, the intensified PH-dependent rises in POXC and WEOC throughout aggregate fractions signify the amplified microbial activities and organic substance turnover in less acidic conditions. POXC, which can be regarded as a sensitive marker of biologically active carbon,^51^ was responding significantly to rising pH, especially in MACA and MESA. This suggests that the liming of legacy effects not only stabilize carbon physically but also promotes biologically mediated carbon cycling. The WEOC permanent concentration is higher in the MICA, which means that smaller aggregates play the role of hotspots of soluble and available carbon.^52^ Although this pool is more susceptible to mineralization, the growth increase with increasing pH might indicate more root exudation and a more extensive microbial by-product. Lower labile carbon pools observed at pH 3.82 therefore suggest a biologically depleted stage of re-acidification characterized by reduced microbial turnover and diminished fresh carbon inputs. Notably, a combination of increased labile carbon fractions along with increased aggregate stability implies a harmonious system of carbon inputs and protection systems work together, opposed to requiring faster degeneration of SOC. Correspondingly, the former authors have mentioned that POXC might be moderated by the soil pH, where pH influences soil chemical characteristics and microbial life, which also influence carbon processes.^53,54^

We found that TN and TP increased with the soil pH and the ultimate TN and TP concentrations at the pH 5.52 could be attributed to better microbial activity, decreased Al^3+^ toxicity,^55^ and increased nutrient retention in stable aggregates. This nutrient distribution pattern further supports the interpretation of pH levels as stages of re-acidification, where nutrient retention capacity progressively declines as soils become more acidic. The preferential accumulation of TN in finer fractions (MICA and SCF) is attributed to good association with organic matter and mineral surfaces, which are very important in long-term conservation of nutrients. Phosphorus exhibited a comparatively constant distribution among aggregate fractions, but always greater concentrations at high PH. This trend is characterized by a decrease of phosphorous fixation by Al and Fe oxides in the less acidic environment and enhanced retention of phosphorus that is bound to organic matters.^49^ Therefore, the advanced re-acidified stage (pH 3.82) likely represents a condition where nutrient immobilization by Al and Fe becomes increasingly dominant, reducing nutrient availability and soil fertility. These results highlight the point that the effects of liming are also present beyond carbon to other parameters of soil fertility. Our results are also supported by previous studies, where MICA and particularly SCF are primarily responsible for the long-term stabilization of organic carbon, N, and a considerable proportion of P is usually facilitated by increased pH levels, which stabilize the soil structure and increase nutrient retention.^56,57^ Additionally,^37^ also indicated that the protracted fertilization methods cause alterations to the soil PH and enhance the nitrogen and phosphorus levels in the soil aggregates.

The findings prove that historical liming has the capacity to cause permanent changes in the structure of soils and in the storage of carbon, even in the absence of more lime added after a long period of time. The benefits are, however, not permanent as evidenced by the partial re-acidification that is seen when liming ceases, and these benefits may be lost over time. The shift from pH 5.52 toward 3.82 reflects a gradual degradation of aggregate stability, carbon protection, biological activity, and nutrient retention during re-acidification. Thus, it is necessary to have balanced and adaptive liming strategies to preserve the ecological stability of the soil without causing excessive disturbance and prompt mineralization of carbon. The stability of aggregate and the preservation of SOC and nutrient retention seem to be heavily dependent on the pH of soil being maintained at an optimal level in Retisols. The results can be used to make meaningful contributions to designing long-term soil management practices to reduce climate change and obtain sustainable agricultural productivity.

## 5 Conclusion

This study demonstrates that the legacy of 75 years of contrasting liming histories continues to influence aggregate stability, carbon turnover, and nutrient retention in Retisols, even after 19 years without lime application. We found that soil carbon pools responded positively to higher pH levels, with maximum soil organic carbon (22.95 g kg^−1^) observed in SCF at 0–10 cm, and permanganate-oxidizable carbon increasing by 54.66% in macro-aggregates compared with intensely acidic soil (pH 3.82). However, water-extractable organic carbon recorded the highest in microaggregates with pH 5.52 under both soil depths. Concurrently, total nitrogen and phosphorus also increased consistently across aggregate fractions with increasing pH level from 3.82 to 5.52. Collectively, raising soil pH to moderately acidic levels substantially strengthened aggregation hierarchy and promoted carbon and nutrient stabilization in Retisols, highlighting the long-term benefits of liming for soil structural resilience and fertility.

### 5.1 Practical management recommendations

The present findings demonstrate that long-term maintenance of moderately acidic soil conditions is essential for sustaining aggregate stability, carbon sequestration, and nutrient retention in Retisols subjected to progressive re-acidification. Based on the observed decline in soil pH following the cessation of liming from 2005 to 2024, periodic maintenance liming is necessary to preserve the structural and biochemical benefits generated by historical liming.

To sustain favorable soil physical conditions and aggregate-associated carbon stabilization, soil pH should be maintained within the range of 5.2 to 5.8. Soil pH values below 5.0 were associated with reduced MACA formation, lower aggregate stability indices, and substantial declines in SOC, POXC, TN, and TP contents. Therefore, preventing further re-acidification below this threshold is critical for maintaining long-term soil resilience.

Under Retisol conditions comparable to those of the present study, maintenance liming at intervals of approximately 5 to 7 years is recommended to counteract progressive soil acidification. However, in high-rainfall environments or under intensive cropping systems where nutrient leaching and acidification rates are accelerated, shorter liming intervals of 4 to 5 years may be more appropriate. Regular soil pH monitoring at 2 to 3 years intervals is further recommended to facilitate timely lime application before severe structural degradation occurs.

Moderate and adaptive liming strategies should be preferred over excessive lime application to avoid abrupt increases in microbial mineralization and potential carbon losses. Surface application combined with shallow incorporation (7–12 cm) appears suitable for improving aggregate stabilization within the biologically active topsoil layer. Furthermore, integrating liming with residue retention, cover cropping, and reduced tillage practices may further enhance MACA formation and long-term carbon protection.

## Conflicts of interest

There are no conflicts to declare.

## Supplementary Material

RA-OLF-D6RA01955B-s001

## Data Availability

All datasets generated and analysed during the current study are available in the manuscript and its supplementary information (SI). Supplementary information is available. See DOI: https://doi.org/10.1039/d6ra01955b.
